# Present-day uplift of the western Alps

**DOI:** 10.1038/srep28404

**Published:** 2016-06-27

**Authors:** J.-M. Nocquet, C. Sue, A. Walpersdorf, T. Tran, N. Lenôtre, P. Vernant, M. Cushing, F. Jouanne, F. Masson, S. Baize, J. Chéry, P. A. van der Beek

**Affiliations:** 1 Geoazur, IRD, Université de Nice Sophia-Antipolis, Observatoire de la Côte d’Azur, CNRS, 250, rue A. Einstein, Valbonne, 06560, France; 2 Chrono-Environnement, CNRS, Univ. de Bourgogne Franche-Comté, 16, route de Gray, 25030 Besançon, Cedex, France; 3 Univ. Grenoble Alpes, ISTerre, F-38000, Grenoble, France; 4 Aquitaine Agency, Bureau de Recherches Géologiques et Minières, 24 avenue Léonard de Vinci, 33600 Pessac, France; 5 Géosciences Montpellier, CNRS-Université Montpellier 2, Montpellier 34095, France; 6 IRSN/PRP-DGE/SCAN/BERSSIN, BP 17, 92262 Fontenay-aux-Roses Cedex, France; 7 Université de Savoie Mont Blanc, ISTerre, 73376 Le Bourget-du-Lac, France; 8 Institut de Physique du Globe de Strasbourg, Université de Strasbourg/EOST, UMR7516, CNRS, France

## Abstract

Collisional mountain belts grow as a consequence of continental plate convergence and eventually disappear under the combined effects of gravitational collapse and erosion. Using a decade of GPS data, we show that the western Alps are currently characterized by zero horizontal velocity boundary conditions, offering the opportunity to investigate orogen evolution at the time of cessation of plate convergence. We find no significant horizontal motion within the belt, but GPS and levelling measurements independently show a regional pattern of uplift reaching ~2.5 mm/yr in the northwestern Alps. Unless a low viscosity crustal root under the northwestern Alps locally enhances the vertical response to surface unloading, the summed effects of isostatic responses to erosion and glaciation explain at most 60% of the observed uplift rates. Rock-uplift rates corrected from transient glacial isostatic adjustment contributions likely exceed erosion rates in the northwestern Alps. In the absence of active convergence, the observed surface uplift must result from deep-seated processes.

The western Alps, the highest topography of Europe, formed during Oligocene-Miocene times, as a consequence of the convergence and indentation of the Adriatic microplate toward Europe^1^. As much as 280 km of shortening was accommodated by underthrusting of the European margin beneath the Adriatic microplate^1^,^2^,^3^. This process resulted in crustal thickening, nappe stacking, and exhumation of crystalline basement, contributing to the building of the present-day topography. During the last million year, the end of active subduction in the Apennines^4^ led to a change of the regional plate-kinematics, with the progressive development of extension in the core of the Apennines and counter-clockwise rotation of the Adriatic micro-plate^5^. A decade of continuous GPS measurements show that, with respect to the Alpine foreland, the GPS sites in the western Po plain have an averaged residual motion of 0.1 mm/yr and provide an upper bound of 0.3 mm/yr (95% confidence level) for possible right-lateral strike slip motion across the western Alps (Supplementary Information). Similarly, south of the western Alps, sites in Corsica and Sardinia show less than 0.4 mm/yr of shortening with respect to the southern western Alps^6^. Therefore, the western Alps is presently a mountain range with virtually zero horizontal velocity boundary conditions, offering a unique opportunity to evaluate the contribution of processes unrelated to horizontal tectonics to the evolution of orogens.

In addition to continuous GPS data, we use levelling data spanning a century in order to study the present-day vertical motion in the western Alps and its surroundings. We show in the Supplementary Information that both data sets have an average internal precision of 0.2 mm/yr and are mutually consistent at a level of 0.3 mm/yr over the studied area. GPS sites located west of the Alpine foreland all show zero vertical rates (±0.2 mm/yr) and therefore define a stable reference frame that we use to express regional vertical rates.

With respect to that reference frame, both GPS and levelling data independently show the following patterns (Fig. 1; Fig. 2a–c): (1) a 80 × 60 km^2^ wide area shows uplift rates exceeding 2 mm/yr in the core of the northwestern Alps (Mont-Blanc and Vanoise massifs), decreasing outward; (2) the southern western Alps (Pelvoux, Queyras and Mercantour massifs) show significantly smaller rates of uplift of the order of 0.5 mm/yr; (3) subsidence reaching 1 mm/yr is observed in the Rhone delta. GPS data further show subsidence rates in the Po plain up to 1.5 mm/yr, increasing eastward^7^.

Unlike horizontal motion that is predominantly controlled by tectonic processes, vertical rates may result from highly diverse mechanisms. At the centennial to millennial time scale, the retreat of Alpine glaciers since the Last Glacial Maximum (LGM) unloads the crust and induces visco-elastic relaxation of the crust-mantle system, still influencing the present-day vertical rates. Modelling results of the Glacial Isostatic Adjustment (GIA) strongly depends on the chosen rheology, the ice cap thickness at LGM and its melting history, all parameters having large uncertainties. In the western Alps, most GIA models predict less than ~0.3 mm/yr of present-day uplift of the Alpine range with respect to its foreland^8^,^9^, but values reaching 1.8 mm/yr have recently been proposed for a 2D model with a low viscosity (10^21^ Pa.s) crustal root beneath the northwestern Alps^10^. At the centennial scale, visco-elastic mid-crustal relaxation induced by the shrinkage of glaciers since the Little Ice Age (LIA) induces a small 0.1–0.2 mm/yr regional signal in the western Alps^11^. Finally, at the decadal scale, the elastic response to a water-equivalent loss rate of nearly 1 m/yr for glaciers in the French western Alps induces a small regional uplift of 0.2 mm/yr but locally reaching 0.9 mm/yr in the Mont-Blanc area^11^. Depending on the model, the summed effects of GIA contributions to the present-day vertical velocities yield a regional signal in the northwestern Alps of ~0.5 mm/yr, possibly reaching ~2 mm/yr in the areas of the largest glaciers (Fig. 2d).

At the million-year timescale, erosion removes rocks from the inner, higher part of mountain ranges, transports the materials by glaciers and rivers, and deposits them in sedimentary basins and major delta fans surrounding the orogen. The response to this mass redistribution implies rock uplift and regional surface height lowering in the erosional areas and rock subsidence and regional surface height increase where sediments load the crust. The amplitude of the response to unloading depends on the density contrast between the crust and the mantle, the characteristic size of the load perturbation and the effective elastic thickness of the lithosphere^12^, but rock uplift reaching 60–80% of the surface unloading are typical values for mountain belts^13^.

It has been proposed that the average sediment discharge from the western Alps to their surrounding basins increased by a factor of two since 5 Myr and a factor of three during the last Myr compared to its constant averaged value between 12 Myr and 5 Myr ago^14^ . The related sedimentary discharge for the last Myr has been estimated to be 4.8 × 10^5 ^km^3^/Myr with an uncertainty of 25% for the western Alps^13^,^14^, a value consistent with estimates of excavation of glacial valleys from geophysical relief as well as independent high-resolution thermochronology data^15^,^16^. This sediment flux together with geophysical relief 17 was used to derive the spatial distribution of rock uplift for the western Alps assuming an elastic response to erosional unloading since 1 Myr^13^. Under the assumption that the erosion rate is the same at present as during the last Myr, the model predicts uplift rates of 0.3–0.5 mm/yr within the western Alps (Fig. 2e) with the highest rates being located in the core of the northern part of the belt. Although the pattern of predicted uplift is qualitatively similar to the geodetic results (Fig. 2a–c,e), Myr-scale erosion explains only 50% of the present-day regional uplift and 30% of the present-day high vertical rate in the northwestern Alps.

Large fluctuations in erosion and sedimentation rates are expected to occur between glaciations and inter-glacial periods, the highest rates being expected during the deglaciation period^18^. As a consequence, it is uncertain whether Myr-scale averaged erosion rates can be used to predict the present-day geodetic rates. At the millennial-scale (~0.5–2.2 kyr), catchment-wide erosion rates derived from *in situ* produced cosmogenic ^10^Be independently indicate erosion rates in the range 0.27–1.33 mm/yr in the core of the western Alps^19^,^20^. Such estimates lead to an average erosion rate of ~0.5 mm/yr similar or slightly higher than Myr-scale average estimates. Finally, at the decadal scale, erosion rates estimated from river loads and reservoir fills indicate a mean erosion rate of 0.32 mm/yr at the scale of the European Alps, reaching 0.58 mm/yr in the northwestern Alps 30% lower than the estimate derived from cosmogenic ^10^Be estimates for the same area^21^. Thus, there is no evidence that present-day erosion rates are significantly higher than their Myr-scale estimates. The discharge rate estimates during the last Myr for the Rhone fan and the Po plain are 1.1 × 10^4^ and 1.3 × 10^4 ^km^3^/Myr respectively^14^. Taking an area of ~10^5 ^km^2^ for the Rhone fan yields a sedimentation rate of ~1.1 mm/yr and a resulting isostatic subsidence rate of ~0.7–0.9 mm/yr. This estimate is in agreement with the subsidence rates found in and around the Rhone delta observed from both levelling and GPS data, within their respective uncertainties. For the Po plain, taking an area of 5 × 10^4 ^km^2^, the same calculation leads to a sedimentation rate of ~2.6 mm/yr. This value is an average over the Po basin, but sedimentation rates are about 3 times larger in the eastern Po plain than in the west^14^. As a consequence, the expected subsidence rate induced by sediment loading is expected to increase from ~1 mm/yr in the western part of the Po plain to ~2 mm/yr in its central part and 3.5–4.0 mm/yr in its easternmost part. These values are again in good agreement with our GPS observations for the western Po plain and also with GPS-derived subsidence rates derived for the eastern Po plain^7^. These simple comparisons suggest that proposed Myr-scale erosion/sedimentation budgets are reasonable first-order estimates of the present-day erosion rates.

Figure 2g,h show the residual GPS and levelling rates once corrected for all surface-process contributions (erosion and GIA) according to previously published models at the scale of the western Alps. While the summed contributions show a similar pattern to the geodetically observed rock-uplift map, the isostatic response to erosion and GIA only explain at most 60% of the highest uplift rates in the central and northern western Alps. The long-term surface uplift rate, defined as the difference between rock uplift rate corrected from GIA transient contributions and erosion rate determines the amount of work against gravity within the orogen^22^. Unless erosion rates and/or GIA contributions would have been underestimated by a factor of at least two, our results suggest that surface uplift is positive at least in the northwestern Alps. Since the surface uplift rates are several times larger than horizontal velocities, they must result from upward tractions applied at the base of the Alpine lithosphere. This hypothesis is consistent with the analysis of gravimetric anomalies in the western Alps, which significantly depart from those predicted by the isostatic equilibrium or flexural elastic support of the plate^23^. As a consequence, the present-day topography is supported dynamically by more buoyant material underneath the range, also possibly promoting the present-day surface uplift.

Tomographic studies in the Alps support that view, as they image a large low velocity anomaly between depths of 90 and 150 km in the northwestern Alps^24^,^25^. Figure 3 shows that the low velocity anomaly overlies a high velocity anomaly interpreted as a detached remnant of the European oceanic lithosphere^24^. There is a clear spatial correlation between the observed uplift pattern and the low-velocity zone both across and along the axis of the western Alpine arc. The replacement of dense by more buoyant material is consistent with normal upward traction at the base of the lithosphere that would induce uplift^8^,^26^. Furthermore, the development of a warm mantle anomaly underneath the western Alps is also expected to promote uplift through a progressive reduction of the equivalent elastic thickness (EET) of the lithosphere and low viscosity crustal root, then enhancing the surface response to deep buoyant material and surface related processes^10^,^27^.

Our geodetic results contradict several previously proposed models for the current deformation regime of the western Alps. Within the western Alps, the average horizontal velocities are 0.26 mm/yr (Supplementary Information). Thus, surface uplift rates in the northwestern Alps are one order of magnitude larger than horizontal velocities. The large ratio of uplift to horizontal rates rules out horizontal motions as the primary control on the present-day deformation in the western Alps^28^. It also excludes the hypothesis of crustal- or lithospheric-scale collapse of the mountain range^29^ which would imply crustal thinning and surface lowering, rather than the observed surface uplift. The crustal state of stress within the western Alps is characterized by widespread extension radial to the belt as indicated by normal faults, extensional focal mechanisms, local geodetic studies^30^,^31^, and extension seen in our GPS solution (Fig. 1b). The areas of extension appear to correlate with the most rapidly uplifting areas (Fig. 2i). Normal faulting would therefore contribute to accommodate the surface uplift-rate gradient driven by the deep dynamics and enhanced by erosion^32^, rather than large-scale gravitational collapse.

The western Alps illustrate a case of early post-collisional evolution. Assuming that the present-day boundary conditions have been set in place ca. 1 Myr ago^4^, our geodetic results do not detect any onset of lithospheric collapse. Although centennial-millennial timescale transient contributions of GIA cannot be ruled out as the dominant forcing to the present-day observed uplift, the good spatial correlation between geodetic results and exhumation rates^25^ advocates for processes persistent at the million year time scale. Erosion and asthenosphere-lithosphere interactions are therefore the processes controlling the longer-term orogen evolution in the absence of relative motion at the boundaries of the belt. In the western Alps, erosion, probably enhanced since mid-Pleistocene times^15^, carves the valleys and increases the height of topographic peaks, but tends to lower the average surface regionally. Mantle-lithosphere interactions counteract and currently exceed this effect in the northwestern Alps. Our geodetic results suggest that orogens can still be growing in the aftermath of the collision stage.

## Methods

The GPS data used in the study are freely available from http://renag.resif.fr and ftp://renag.unice.fr. The levelling data are subject to prior agreement from the IRSN and BRGM (edward.cushing@irsn.fr, n.lenotre@brgm.fr). GPS and levelling analysis codes are available on request from JMN. The GPS and levelling analysis are fully described in the Supplementary Information.

## Additional Information

**How to cite this article**: Nocquet, J.-M. *et al*. Present-day uplift of the western Alps. *Sci. Rep*. **6**, 28404; doi: 10.1038/srep28404 (2016).

## Supplementary Material

Supplementary Information

## Figures and Tables

**Figure 1:**
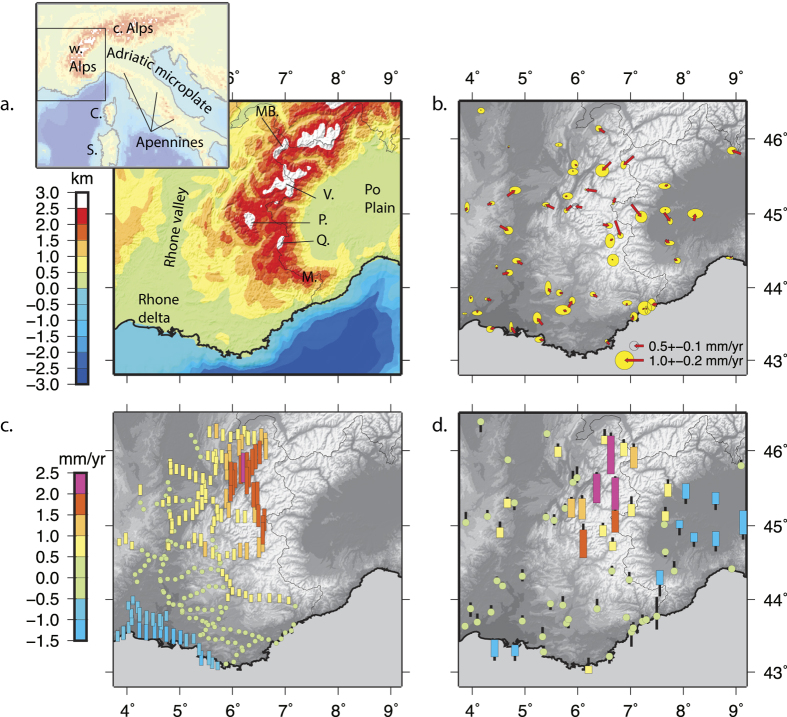
Location map and geodetic results. (**a**) Location map. Colour indicates the topography averaged using a 25 km-width Gaussian filter. MB: Mont Blanc massif. V: Vanoise massif. P: Pelvoux massif. Q: Queyras massif. M: Mercantour massif. (**b**) GPS Horizontal GPS velocity field. Error ellipses are at the 95% confidence level. (**c**) Adjusted levelling rates. Data are decimated every 10 km. (**d**) GPS vertical velocities. Black lines indicate 1-σ uncertainties. Orange, red and purple bars indicate uplift and blue ones subsidence. Levelling and GPS data are with respect to the Alpine foreland as described in the main text. Figure created with GMT v. 5.1 (http://gmt.soest.hawaii.edu/).

**Figure 2:**
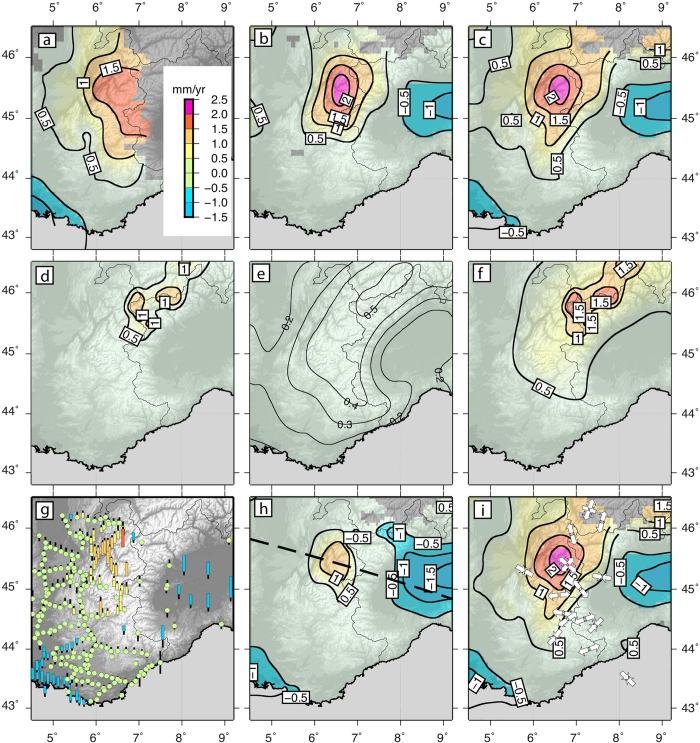
Vertical rate maps and models predictions. (**a**) Vertical rate map from levelling data. (**b**) Vertical rate map from GPS data. (**c**) Levelling-GPS combined map of vertical rate. (**d**) Predicted uplift-rate map from all GIA contributions^9^,^11^. (**e**) Uplift-rate map predicted from the response to erosion^11^. (**f**) Predicted uplift-rate map from the sum of GIA and erosion contributions. (**g**) Residual geodetic rates once corrected for GIA and erosion contributions. (**h**) Same as g in map view. (**i**) Seismotectonic stress tensors^28^ superimposed on Fig. 2c. Dashed line in panel h indicates the location of the profile shown in Fig. 3. Figure created with GMT v. 5.1 (http://gmt.soest.hawaii.edu/).

**Figure 3:**
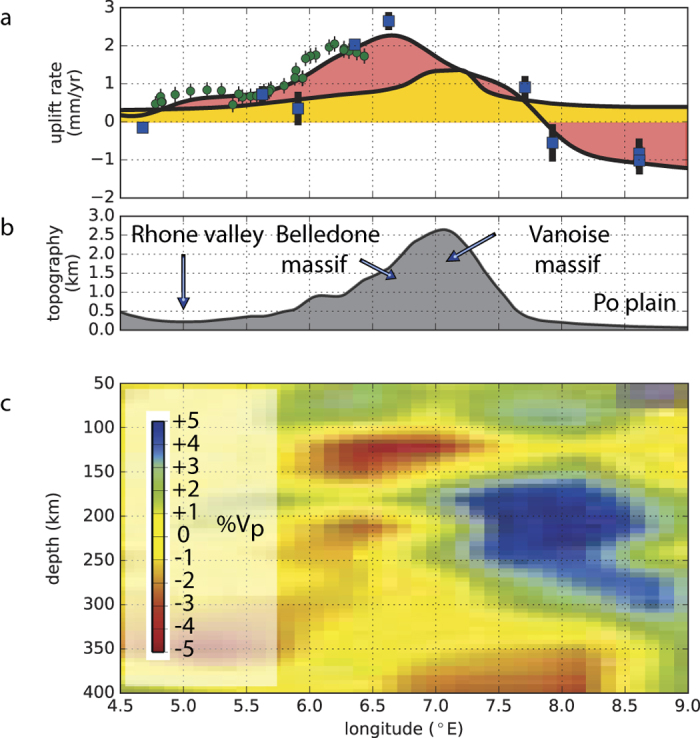
Cross-section of vertical rates and deep structure beneath the northwestern Alps. Cross-section location is shown in Fig. 2h. (**a**) Uplift rates. Green and blue dots are levelling and GPS rates respectively, with error bars (95% confidence level) indicated by the thin black vertical lines. The pink-coloured fill shows the GPS-levelling combined uplift-rate profile from Fig. 2c. The yellow fill shows the summed prediction of GIA and erosion models from Fig. 2f. (**b**) Topography along the cross section averaged using a 50 km-width Gaussian filter. (**c**) Upper mantle tomographic model from ref. 24. Figure created using Matplotlib v. 1.4 (http://matplotlib.org).
