# Supplementary material for: Present-day uplift of the western Alps
Source: Sci Rep. 2016 Jun 27;6:28404. doi: 10.1038/srep28404 (PMC4921835; doi:10.1038/srep28404)
Supplement: Supplementary Information [file srep28404-s1.pdf]

Supplementary Information for the paper
"Present-day uplift of the western Alps"

by J.-M. Nocquet, C. Sue, A. Walpersdorf, T. Tran, N. Lenôtre, P. Vernant, M. Cushing, F. Jouanne, F. Masson, S. Baize, J. Chéry, P. A. van der Beek

GPS analysis

Data set & processing strategy

The Continuous GPS data used in this study are from the RENAG network (http://webrenag.unice.fr), from the RGP network (http://rgp.ign.fr), from the ORPHEON network (http://reseau-orpheon.fr), from the Piemonte region (http://gnss.regione.piemonte.it), from the RING network from the Istituto Nazionale di Geofisica e Vulcanologia (INGV, http://ring.gm.ingv.it) and from EUREF (http://www.epncb.oma.be). The period 2002.0-2013.5 was analyzed using the GAMIT/GLOBK 10.5 software package [1]. The analysis is divided into 3 steps: (1) raw phase and pseudo-range observations are reduced to produce daily loosely constrained solutions (2) loosely constrained daily solutions are expressed in a consistent reference frame using a Helmert transformation to obtain daily positions time series (3) time series are analyzed in order to detect outliers and offsets. The corrected and cleaned time series are used to assess the noise properties and finally derive the velocities estimates with their associated uncertainties.

For the first step, we use the final combined orbits from the International GNSS Service for Geodynamics [2] derived from the first reprocessing campaign (http://acc.igs.org/reprocess.html). Because the orbits were reprocessed using a consistent strategy for the whole period, they were taken as fixed in the processing. We use the ocean loading correction model FES2004 computed from the tidal hydrodynamic equations and data assimilation [3]. We use the the Vienna Mapping Function (VMF1, [4]) for both hydrostatic and nonhydrostatic models of the tropospheric delay and the global pressure and temperature model. We estimate a zenithal trophospheric delay parameter every 2 hours and two horizontal atmospheric gradient per day as stochastic parameters. We also correct for atmospheric tides and atmospheric loading. The atmospheric loading corrections used together with the VMF1 mapping functions have proven to provide the best results in terms of repeatabilities and noise characteristics [5]. Finally, IGS absolute calibration of Phase Center Variations (PCV) of the antennas have also been used, as recommended by the IGS.

Our second step differs from most used strategies. Instead of expressing our solution in the current ITRF [6] and then try to remove the regional common mode motion, we start by identifying a subset of sites with good repeatabilities and little loss of data over the studied time window. We find that CGPS sites SOPH, MTPL, SJDV, ZIMM and GENO meet such criteria. We then used this subset of sites to define a local reference frame using the following strategy: (1) in a first run, we use their coordinates defined in the ITRF2008 at epoch 2007.0 [6] with 0 velocity as the a priori reference frame and estimate a 7-parameters transformation to derive a first solution (2) then, we estimate the new position at the reference epoch and velocity for the chosen subset of sites, defining the reference frame for the next iteration (3) this process

is iterated until estimated positions and velocities agree with the reference frame at the level of 0.1 m for positions and 0.01 mm/yr for velocities. Compared with the classical methodology that uses the raw ITRF as the reference frame, our approach improves the repeatability by a factor of 2, removes the regional common mode signal and improves the noise characteristics. Furthermore, this approach is more rigorous than the classical ITRF/common mode estimation in the sense that the true shape of the network is preserved throughout the procedure because we use only 7-parameters transformations. As a consequence, the obtained results only reflect the change of the network shape through time.

In a third step, we used the approach proposed by [7] and implemented in the CATS software [8] to assess the noise characteristics and estimate offsets, velocity, and amplitude of seasonal variations, together with the associated uncertainties.

Results & precision

GPS results are provided in Supplementary Information Table 1. Extended Data Figure 1 shows the time series for a selection of sites. Long-term repeatabilities on the vertical component are found in the range of 2.0 to 4.5 mm. As for most GPS time series, we find that a combination of white and flicker noise best explains the noise characteristics on the GPS time series [9]. Extended Data Figure 2 shows the histogram of the spectral index κ characterizing the noise when estimated through the Maximum Likehood Estimator embedded in CATS. It shows that $-1.6 < \kappa < -0.4$, with a median value of -0.7 for the verticall component. As a result of this analysis, formal errors on velocity components are of the order of a few tenths of mm/yr (0.1-0.6 mm/yr), with the best behaving sites having a formal error of 0.1 mm/yr. Although these formal uncertainties might still be over-optimistic, further external criteria described below indicate that main trends of the vertical velocity field can be trusted at the 0.3 mm/yr level.

Kinematic conditions at the boundaries of the western Alps

We use the best determined sites in our solution to estimate the relative motion between sites located west of the Alpine foreland in France and sites located in the westernmost part of the Po plain in Italy. We calculate the Euler pole for the sites located east of the western Alps with respect to the sites located west of the Alps(Table 3). We do not detect any internal deformation in the western Po plain, as indicated by a wrms of 0.07 mm/yr. The wrms for the Alpine foreland in France is 0.05 mm/yr. The relative pole between the two domains predicts 0.1-0.2 mm/yr of strike-slip motion between the western part of the Adriatic plate and the stable foreland of the western Alps in France (Figure 1a of the main text). Accounting for the Euler pole uncertainty, we therefore find that 0.3 mm/yr is a strict upper bound of possible motion the western Po plain. Because at this level of precision we might reach the limitation of idealized rigid micro-blocks, we also used the more direct observation of relative velocities between sites west of the Alpine foreland and the western Po plain (Supplementary Table 2). Individual baselines show that the relative motion average over the sites is $<$ 0.1mm/yr. No velocity $>$ 0.25 mm/yr is found between pairs of sites, confirming 0.3 mm/yr as an upper bound for kinematic boundary conditions of the western Alps.

Internal horizontal deformation within the western Alps

We calculate an Euler Pole for the sites located within the Alps and its surroundings and within the Alps only. The first calculation leads to a wrms=0.23 mm/yr and the second to wrms=0. 26 mm/yr, indicating a very small amount of possible horizontal deformation within the western Alps. The largest residuals are found in the northwestern Alps close to the area of maximum uplift. As indicated by Table 4, residuals are found to be statistically highly significant for sites CHTL, JANU, MODA, PUYA, GRAV, LFAZ, VILR, MRGE. Using the sites located in the area of significant uplift, we find that the deformation is primarly extensionnal at a ~20 nstrain/yr are in a N120° direction (Table 5)

Stable Alpine foreland vertical reference frame

As for the horizontal velocities, we want the vertical reference frame to be relevant for the tectonic processes studied here. We therefore discard the use of the ITRF which realizes a center of the mass for the whole Earth frame. Since our study focuses on the Alps dynamics, we choose to define a reference frame outside of

the Alps and test its stability. Around the Alps, the sites having the best determined vertical velocities are (MTPL, GENO, SJDV, GRAS). They show a level of internal agreement of 0.13 mm/yr. With respect to this reference frame, all sites suROUNDING the western Alps with a vertical velocity uncertainty $\sigma_{up} < 0.3$ mm/yr agree within 0.21 mm/yr (wrms). The vertical rate averaged over (MTPL, GENO, SJDV, GRAS) therefore provide a stable vertical reference frame, with no internal vertical deformation at the precision of the data and making sense from a tectonic point of view. The chosen reference frame certainly does no cause any bias larger than 0.2 mm/yr in the vertical rates seen in the western Alps.

Levelling data & adjustment

Data set

Levelling data are from the National Geographic Institute of France (IGN, http://www.ign.fr), partly analyzed in [10, 11, 12]. For each profile i and levelling benchmark j, data were provided as height differences Δz_j^i between the two epochs of measurements with respect to a reference benchmark of each profile i following the methodology described in [13]. The time span between two levelling surveys ranges from 58.25 to 112.83 years. Assuming constant velocity, the levelling input data set prior adjustment is $\Delta v_j^i = \frac{\Delta z_j^i}{\Delta t^i}$, where Δt^i is the time span between two successive surveys of profile i. We used 24 levelling profiles covering the western Alps and its foreland in France (Extended Data Figure 3 & Extended Data Figure 4), including a total of 1655 benchmarks, with an average distance between benckmarks of ~2km. Details of the levelling protocol for both periods of surveys are described in [14] and [11].

Combined levelling-GPS adjustment model

We use a combined least-squares adjustment to derive a vertical velocity field including levelling profiles and GPS vertical velocities. The adjustment model relates the vertical velocity v_j^i of each levelling benchmark j from profile i to the observed velocity difference $\mathbf{\Delta v_j^i}$ and nearby GPS vertical velocity $\mathbf{v_{GPS_j^i}}$:

$$v_j^i - v_0^i = \mathbf{\Delta v_j^i} \; (\sigma_j^i) \tag{1}$$

$$v_j^i = v_l^k \; (\sigma_{jl}^{ik}) \tag{2}$$

$$v_j^i = \mathbf{v_{GPS_j^i}} \; (\sigma_j^{i-GPS_j^i}) \tag{3}$$

Equation 1 is simply the levelling observation equation. The weight associated to this equation is $1/(\sigma_j^i)^2$, where σ_j^i is chosen according to the specification provided the IGN. Misclosure of levelling loops indicate a relative standard deviation of 2.6 $mm/\sqrt{km}$ for the ancient surveys (1886-1907). For the more recent surveys (1965-1979), misclosures indicate a formal error of 1.8 $mm/\sqrt{km}$ and 2.6 $mm/\sqrt{km}$ for the first and second-order loop respectively. We conservately chose a value of 2.6 $mm/\sqrt{km}$ for all surveys and define σ_j^i by

$$\sigma_j^i = \frac{\sqrt{2 * 2.6^2}}{\Delta t^i} \sqrt{d^i(j,0)} \tag{4}$$

where $d^i(j,0)$ is the curved distance between benchmark j and the reference benchmark of profile i. This formula gives an average model error of 0.037 $mm/yr/\sqrt{km}$.

Equation 2 is a condition equation constraining the vertical velocities to be equal (within a given uncertainty) at sites common to two levelling profiles i and k. Because we want to avoid any error on a common site to propagate into the adjustment, and because the vertical velocity field is assummed to be smooth at the scale of a few km, we used equation 2 not only at strictly common sites, but at all sites within a certain radius (typically 5 or 10 km), with the weight decreasing with distance d betwwen the two levelling benchmarks:

$$\sigma_{jl}^{ik} = \sqrt{\frac{\sigma_0^2}{e^{-d_{jl}/d_c}}} \tag{5}$$

where σ_0 is a reference error value, d_{jl} is the distance between benchmark j from profile k and benchmark l from profile i and d_c a critical distance to impose the constraint. In order to choose σ_0 and d_c, we checked the WRMS at each area where profiles cross.

Equation 3 is a condition equation constraining the velocity to be equal (again, within a given uncertainty) between a levelling site with velocity v_j^i and the uplift rate of a nearby GPS site $v_{GPS_j^i}$. Here the weight of the constraint is given by:

$$\sigma^{j-GPS_j^i} = \sqrt{\frac{\sigma^2_{GPS_j^i} + \sigma^2_{GPS}}{e^{-d/d_c}}} \tag{6}$$

where d is now the distance between the levelling benchmark and the nearby GPS site, σ_{GPS} the uncertainty of the GPS vertical velocity, and $\sigma_{GPS_j^i}$ a reference level of constraint for levelling-GPS velocities.

The chosen adjustment model offers the versatility to test different constraints for levelling-levelling data and GPS-levelling data, and search for outliers. It is further useful to express the levelling results in the GPS reference frame previously defined. Levelling results are often shown with respect to a fixed reference point and the obtained results are therefore highly dependent on the behaviour of this site. Here, equation 3 allows to express levelling results in the same reference (that is average in the least-squares sense) as the GPS results. This is optimal because, on the contrary to levelling results that can suffer from long length scale biases [15], GPS accuracy is largely independent from the scale of the network. Furthermore, we show in the previous paragraph that we could define a tectonically relevant and stable vertical reference frame that we use to express all results.

Levelling adjustment results

We use the previous combination model, with the very weak constraint σ_{GPS}=1 000 mm/yr for GPS-levelling equality condition. This level of constraint is sufficient to ensure that levelling adjusted velocities are expressed in the same reference frame as the GPS results, without inducing any distortion of the intrinsic information provided by the levelling measurements.

We find that $\sigma_0 = 0.2$ mm/yr and $d_c = 4$ km provides adjustments with good statistical indicators (histogram of normalized residuals close to the standard normal distribution). Extended Data Figure 5 shows the distribution of residuals. It shows that the average internal consistency among the profiles is ~0.2 mm/yr, indicating that this value can be retained as an average indicator of the precision of the levelling results at the 1σ confidence level or ~0.4 mm/yr at the 95% confidence level.

Combined GPS-levelling adjustment results - Accuracy estimates

The combined GPS-levelling adjustement has been obtained by progressively increasing the equality constraint of equation 3 by decreasing $\sigma_{GPS_j^i}$. Ideally, if the GPS sites were colocated with levelling benchmarks and GPS and levelling would perfectly agree, we could decrease $\sigma_{GPS_j^i}$ to 0. We fixed d_c to the average distance of GPS to the closest levelling benchmark (20 km) and find that $\sigma_{GPS_j^i} = 0.2$ mm/yr provides residuals for equation 3 that are in agreement with the GPS uncertainties. Nonetheless, the combined adjustment also indicates areas of significant differences that are further enlighted in Figure 1 of the main text. In the Alpine foreland between longitude 5°E (the Rhone valley) and 6°E, levelling data indicate 0.5-1.0 mm/yr uplift rates that are not seen in the GPS data. To a lesser extent, a similar difference is found in the southern Alps. Finally, a larger subsiding area is found in the Rhone delta. The origin of these differences have yet to be understood. While GPS are located on rocks, levelling data are located along roads, often on sediments. Sediment compaction through time could explain the larger subsidence rates seen in the levelling results, but does not explain the 0.5 mm/yr larger levelling uplift rates recorded in the southern Alps and western pre-Alps. There is no systematic correlation with height that could be explained by a systematic bias in GPS or levelling. Given these results, we chose to interpret only (1) patterns that are consistent between the two techniques; (2) we chose 0.5 mm/yr as a threshold for deciding whether a motion is significant or not.

Map of uplift & models

The map of uplift were simply derived by interpolating the data points using the "surface" algorithm embedded in the GMT software [16](http://gmt.soest.hawaii.edu/). We used an L-curve [17] criterion to choose the tension factor providing the smoothest model explaining the data. The wrms for the chosen model is 0.17 mm/yr for the GPS velocities. For the levelling data, the average overall wrms 0.07 mm/yr. wrms reach 0.15-0.23 mm/yr for profiles 193,174,171,200, were the largest levelling-GPS rate differences are observed.

For ensuring that GIA and erosion physical models and geodetic results are in the same vertical reference frame, the models were corrected by the average uplift rates predicted at the GPS sites used to define the vertical reference frame used throughout the study.

References

[1] Herring, T. A., King, R. W., Floyd, M. A. & McClusky, S. C. GAMIT/GLOBK Reference manual, 10.6. Tech. Rep., Massachusetts Institute of Technology (2015).

[2] Dow, J. M., Neilan, R. E. & Rizos, C. The International GNSS Service in a changing landscape of Global Navigation Satellite Systems. *Journal of Geodesy* **83**, 191–198 (2009).

[3] Lyard, F., Lefevre, F., Letellier, T. & Francis, O. Modelling the global ocean tides: Modern insights from FES2004. *Ocean Dynamics* **56**, 394–415 (2006).

[4] Boehm, J., Werl, B. & Schuh, H. Troposphere mapping functions for GPS and very long baseline interferometry from European Centre for Medium-Range Weather Forecasts operational analysis data. *Journal of Geophysical Research* **111**, 1–9 (2006). URL `http://www.agu.org/pubs/crossref/2006/2005JB003629.shtml`.

[5] Tregoning, P. & Watson, C. Atmospheric effects and spurious signals in GPS analyses. *Journal of Geophysical Research* **114**, 1–15 (2009). URL `http://www.agu.org/pubs/crossref/2009/2009JB006344.shtml`.

[6] Altamimi, Z., Collilieux, X. & Métivier, L. ITRF2008: an improved solution of the international terrestrial reference frame. *Journal of Geodesy* **85**, 457–473 (2011). URL `http://www.springerlink.com/index/10.1007/s00190-011-0444-4`.

[7] Williams, S. D. P. The effect of coloured noise on the uncertainties of rates estimated from geodetic time series. *Journal of Geodesy* **76**, 483–494 (2003). URL `http://www.springerlink.com/openurl.asp?genre=article\&id=doi:10.1007/s00190-002-0283-4`.

[8] Williams, S. D. P. CATS: GPS coordinate time series analysis software. *GPS Solutions* **12**, 147–153 (2008).

[9] Williams, S. D. P. Error analysis of continuous GPS position time series. *Journal of Geophysical Research* **109**, 1–19 (2004). URL `http://www.agu.org/pubs/crossref/2004/2003JB002741.shtml`.

[10] Lenôtre, N. Mouvements Verticaux Actuels dans les Alpes - Comparaison de Nivellements. Tech. Rep., BRGM-CEA-IPSN, R 31886 GEO SGN 90 (1990).

[11] Jouanne, F., Ménard, G. & Darmendrail, X. Present-day vertical displacements in the north-western Alps and southern Jura Mountains: Data from leveling comparisons. *Tectonics* **14**, 606 (1995).

[12] Jouanne, F., Genaudeau, N., Ménard, G. & Darmendrail, X. Estimating present-day displacement fields and tectonic deformation in active mountain belts: an example from the Chartreuse Massif and the southern Jura Mountains, western Alps. *Tectonophysics* **296**, 403–419 (1998). URL `http://linkinghub.elsevier.com/retrieve/pii/S0040195198001565`.

[13] Lenôtre, N., Thierry, P., Blanchin, R. & Brochard, G. Current vertical movement demonstrated by comparative levelling in brittany (northwestern france). *Tectonophysics* **301**, 333–344 (1999).

[14] Duquesne, H. & Jeannot, M. Le réseau français de nivellement de précision, préparation à la rédaction du schéma directeur de la géodésie. Tech. Rep., IGN (1989).

[15] Kasser, M. Un nivellement de très haute précision: la traversée marseille-dunkerque 1983. *Comptes rendus de l'Académie des sciences. Série 2, Mécanique, Physique, Chimie, Sciences de l'univers, Sciences de la Terre* **309**, 695–700 (1989).

[16] Wessel, P., Smith, W. H., Scharroo, R., Luis, J. & Wobbe, F. Generic mapping tools: improved version released. *Eos, Transactions American Geophysical Union* **94**, 409–410 (2013).

[17] Hansen, P. C. Analysis of discrete ill-posed problems by means of the l-curve. *SIAM review* **34**, 561–580 (1992).

Supplementary Tables

Site	λ	ϕ	v_e	v_n	v_u	σ_{v_e}	σ_{v_n}	σ_{v_u}	period	days	years	κ_e	κ_n	κ_u
ALES	44.92	8.62	-1.09	0.97	-1.02	0.23	0.13	0.37	2006.0-2011.0	943	5.0	-0.79	-0.46	-0.40
ALPE	45.09	6.08	-0.36	0.07	1.43	0.06	0.06	0.23	2007.0-2013.6	1621	6.6	-0.37	-0.54	-0.56
ANNO	45.24	4.67	0.41	0.29	0.64	0.12	0.08	0.12	2007.5-2013.6	1462	6.1	-1.14	-0.87	-0.32
ASTI	44.91	8.20	0.07	0.38	-0.64	0.16	0.10	0.34	2006.0-2011.0	1365	5.0	-1.07	-0.92	-0.89
AURI	43.71	4.95	0.06	-0.75	0.24	0.11	0.22	0.14	2007.6-2013.6	1341	6.1	-1.15	-1.48	-0.38
AUTN	46.95	4.29	0.09	-0.13	-0.45	0.09	0.15	0.32	2008.0-2013.6	1910	5.6	-0.60	-0.72	-0.58
AXPV	43.49	5.33	-0.29	0.35	0.41	0.08	0.12	0.25	2004.7-2013.6	2701	8.9	-0.91	-1.09	-0.91
BACT	44.39	6.65	0.06	-0.05	0.02	0.11	0.12	0.35	2007.6-2013.6	1990	6.0	-0.90	-0.74	-0.51
BAUB	43.88	3.97	0.02	-0.12	-0.08	0.05	0.07	0.49	2007.9-2013.3	1572	5.4	-0.47	-0.56	-0.90
BIEL	45.56	8.05	0.02	-0.41	-1.05	0.19	0.17	0.35	2006.2-2010.0	1110	3.8	-1.10	-1.16	-0.49
BLIX	43.87	6.37	-0.22	0.14	0.36	0.06	0.05	0.33	2009.3-2013.6	1347	4.3	-0.31	-0.31	-0.50
CAST	45.39	7.71	-0.17	-0.06	0.91	0.12	0.06	0.30	2006.0-2011.0	1286	5.0	-0.94	-0.66	-0.70
CBRY	45.58	5.91	0.00	-0.08	0.35	0.11	0.07	0.44	2007.0-2013.6	2172	6.6	-0.94	-0.73	-1.01
CHAM	45.11	5.88	-0.30	-0.27	1.31	0.04	0.05	0.12	2004.0-2013.6	2559	9.6	-0.67	-0.69	-0.56
CHMX	45.93	6.87	-2.02	-0.65	0.94	0.26	0.19	0.25	2007.7-2013.6	1289	6.0	-0.96	-0.76	-0.48
CHTL	45.30	6.36	-0.58	0.08	2.03	0.02	0.02	0.12	2004.0-2013.6	2208	9.6	-0.31	-0.39	-0.80
CNNS	43.55	7.02	0.14	0.19	-0.24	0.07	0.09	0.78	2008.5-2013.6	1569	5.1	-1.04	-1.11	-1.29
COMO	45.80	9.10	-0.48	0.17	0.28	0.10	0.08	0.13	2007.0-2013.6	1786	6.6	-0.94	-0.90	-0.66
CUNE	44.39	7.55	0.23	0.45	-1.00	0.32	0.26	0.79	2006.0-2011.0	1306	5.0	-1.25	-1.20	-1.13
ESAB	45.31	4.80	0.11	-0.13	-0.18	0.24	0.16	0.32	2008.0-2013.6	1757	5.6	-1.19	-1.12	-0.89
EZEV	43.77	7.50	-0.27	-0.03	0.20	0.08	0.12	1.12	2009.2-2013.6	1428	4.5	-0.64	-1.03	-1.18
FCLZ	45.64	5.99	-0.12	0.07	0.20	0.07	0.09	0.57	2004.0-2007.8	1033	3.8	-0.50	-0.58	-0.72
GENO	44.42	8.92	-0.12	-0.05	-0.13	0.08	0.03	0.06	2004.0-2013.6	2461	9.6	-1.19	-0.80	-0.64
GINA	43.68	5.79	-0.18	0.07	0.01	0.16	0.10	0.28	2004.0-2009.0	1627	5.0	-1.55	-0.93	-0.87
GLRA	44.84	4.52	0.45	-0.24	0.72	0.09	0.08	0.40	2008.1-2013.6	1676	5.5	-0.34	-0.21	-0.75
GRAS	43.75	6.92	0.04	0.14	0.09	0.10	0.05	0.10	2005.1-2013.6	2743	8.5	-1.16	-0.87	-0.67
GRAV	45.13	7.02	0.52	-0.65	0.84	0.12	0.12	0.53	2007.8-2011.0	731	3.2	-0.35	-0.33	-0.43
GROG	43.43	9.89	-0.34	0.47	-0.07	0.04	0.05	0.57	2007.0-2012.8	1742	5.8	-0.47	-0.67	-1.11
GUIL	44.66	6.66	-0.14	-0.10	0.64	0.10	0.14	0.28	2007.9-2013.6	860	5.8	-1.05	-1.25	-0.84
IENG	45.02	7.64	0.32	-0.45	0.23	0.05	0.07	0.29	2007.0-2013.6	2121	6.6	-0.62	-0.61	-0.98
JANU	44.91	6.71	0.28	-0.79	1.52	0.06	0.06	0.45	2007.0-2012.9	749	5.9	-0.51	-0.50	-0.75
LASP	44.07	9.84	-0.10	0.49	0.25	0.04	0.07	0.55	2007.0-2013.6	2112	6.6	-0.81	-0.91	-1.15
LEBE	45.92	5.62	0.04	0.02	0.72	0.07	0.04	0.21	2007.5-2013.6	1720	6.1	-0.95	-0.65	-0.51
LFAZ	45.12	5.40	0.02	-0.03	0.07	0.04	0.03	0.21	2006.0-2013.4	1910	7.4	-0.48	-0.33	-0.73
LUVI	43.69	4.10	-0.01	-0.15	0.38	0.10	0.05	0.20	2007.5-2013.6	1394	6.1	-1.17	-0.68	-0.91
MARG	46.08	6.51	-0.32	0.21	0.60	0.06	0.06	0.45	2007.4-2013.6	2080	6.3	-0.62	-0.53	-0.87
MARS	43.28	5.35	-0.23	0.03	0.20	0.09	0.06	0.23	2004.0-2011.3	2295	7.3	-0.92	-0.55	-0.53
MICH	43.92	5.72	-0.19	0.02	0.47	0.06	0.07	0.15	2004.0-2009.6	1651	5.6	-0.96	-0.93	-1.07
MODA	45.21	6.71	-0.10	-0.28	2.24	0.03	0.03	0.09	2004.0-2013.6	2786	9.6	-0.61	-0.36	-0.44
MONC	45.07	7.93	0.18	-0.06	-0.55	0.19	0.09	0.49	2007.0-2012.8	1957	5.8	-1.35	-1.05	-0.97
MOND	44.39	7.83	0.17	-0.08	0.34	0.07	0.06	0.34	2006.0-2011.0	1024	5.0	-0.78	-0.52	-0.78
MRGE	45.77	7.06	-0.53	-0.46	1.47	0.06	0.08	0.24	2007.0-2013.6	1948	6.6	-0.78	-0.90	-0.56
MTPL	43.64	3.86	-0.02	0.03	0.14	0.05	0.02	0.06	2004.0-2013.6	2932	9.6	-1.05	-0.77	-0.75
NICA	43.70	7.23	0.11	0.06	0.16	0.14	0.14	0.30	2004.0-2013.6	2041	9.6	-1.49	-1.35	-1.22
NICE	43.73	7.30	0.00	-0.10	0.05	0.03	0.07	0.23	2004.0-2013.6	2996	9.6	-0.82	-1.28	-1.18
NIME	43.83	4.36	-0.25	0.07	0.17	0.03	0.03	0.29	2007.1-2013.6	1912	6.5	-0.63	-0.41	-1.02
NOVA	45.45	8.61	-0.56	-0.12	-0.83	0.21	0.08	0.39	2004.0-2011.0	1475	7.0	-1.23	-0.76	-1.02
PALI	43.38	4.81	-0.21	0.33	-0.77	0.04	0.10	0.34	2007.9-2013.6	1316	5.7	-0.63	-1.03	-1.04
PAVI	45.20	9.14	-0.54	0.47	-1.65	0.25	0.12	0.36	2004.0-2011.0	1777	7.0	-0.96	-0.96	-0.83
POBU	46.38	4.16	0.02	-0.05	-0.27	0.07	0.06	0.47	2008.0-2013.6	1683	5.6	-0.79	-0.68	-1.00
PQRL	42.98	6.21	0.53	-0.41	0.52	0.16	0.17	0.57	2006.0-2010.8	1238	4.8	-0.88	-0.95	-0.71
PRNY	46.90	6.34	-0.85	-0.10	0.03	0.66	0.10	0.41	2007.8-2013.6	1980	5.8	-1.39	-0.72	-0.70
PUYA	44.86	6.48	0.33	-0.06	0.77	0.07	0.06	0.17	2006.0-2013.6	2287	7.6	-0.69	-0.88	-0.54
PUYV	45.04	3.88	0.07	0.18	0.40	0.05	0.10	0.33	2004.7-2013.6	2568	8.9	-0.61	-1.16	-1.09
RABU	44.27	6.98	0.28	-0.19	0.12	0.30	0.16	0.41	2004.0-2013.6	1820	9.6	-0.96	-0.93	-0.84
ROSD	45.69	6.63	-0.45	-0.44	2.65	0.13	0.12	0.24	2006.0-2012.4	1754	6.4	-0.83	-0.98	-0.58
RSTL	43.94	5.48	-0.13	0.20	-0.19	0.06	0.14	0.22	2006.0-2013.6	2123	7.6	-0.72	-1.20	-1.06
SAUV	44.26	4.47	-0.02	0.12	0.14	0.03	0.04	0.16	2004.5-2013.6	1581	9.2	-0.68	-0.51	-0.84
SAVI	44.65	7.66	0.22	-0.15	-0.08	0.10	0.05	0.28	2006.0-2010.3	1059	4.3	-0.74	-0.47	-0.72
SEUR	46.99	5.15	0.07	-0.03	0.20	0.05	0.10	0.30	2007.1-2013.6	2034	6.5	-0.54	-1.23	-0.92
SIMA	46.22	5.42	-0.16	0.18	-0.08	0.36	0.32	0.20	2007.6-2013.6	1332	6.0	-1.68	-1.36	-0.44
SJDV	45.88	4.68	0.04	0.07	-0.15	0.02	0.02	0.10	2004.0-2013.6	3023	9.6	-0.53	-0.53	-0.75
SOPH	43.61	7.05	-0.10	-0.01	0.27	0.04	0.03	0.12	2004.0-2013.6	3087	9.6	-0.93	-0.76	-0.98
STEY	45.24	5.76	0.13	0.01	0.11	0.10	0.07	0.21	2004.0-2013.6	2180	9.6	-0.90	-0.99	-0.79
STGR	44.19	4.57	0.35	0.07	0.13	0.08	0.06	0.16	2007.5-2013.6	1499	6.1	-0.69	-0.45	-0.41
STMR	43.45	4.42	-0.23	-0.07	-1.20	0.07	0.07	0.27	2008.3-2013.6	1551	5.3	-1.03	-0.84	-1.01
STV2	44.57	6.11	-0.33	-0.23	1.95	0.15	0.21	0.43	2009.0-2013.6	1233	4.6	-1.11	-0.97	-0.64
TENC	45.12	4.29	0.24	0.08	0.15	0.06	0.04	0.29	2004.5-2013.6	1999	9.1	-0.68	-0.30	-0.95
TORI	45.06	7.66	-0.01	-0.06	0.69	0.11	0.07	0.30	2004.0-2013.2	2539	9.2	-0.96	-0.84	-1.01
TROP	43.22	6.60	-0.16	0.18	-0.40	0.03	0.03	0.11	2006.0-2013.6	1917	7.6	-0.50	-0.81	-0.67
VAUD	46.98	5.63	0.37	0.05	-0.37	0.08	0.09	0.17	2008.1-2013.6	1782	5.5	-0.71	-0.83	-0.50
VILR	45.07	5.55	-0.43	0.21	0.04	0.07	0.04	0.38	2007.0-2013.6	2073	6.6	-0.57	-0.27	-0.61
VINO	43.73	5.82	0.19	0.34	-0.29	0.06	0.07	0.34	2007.5-2013.6	1367	6.1	-0.72	-0.84	-1.05
VISN	44.32	4.95	-0.30	0.18	0.05	0.09	0.06	0.42	2007.6-2013.6	1795	6.0	-0.87	-0.35	-0.77
ZIM2	46.88	7.47	0.08	0.17	1.19	0.03	0.12	0.34	2007.9-2013.6	1551	5.7	-0.38	-1.03	-0.79
ZIMM	46.88	7.47	-0.32	0.34	1.01	0.10	0.06	0.06	2004.0-2013.6	3112	9.6	-1.28	-1.07	-0.52

Table 1: GPS velocities and related information. λ, ϕ: longitude and latitude of GPS sites in decimal degrees. $v_e, v_n, v_u, \sigma_{v_e}, \sigma_{v_n}, \sigma_{v_u}$: estimated values for the east, north and up components respectively, together with the associated uncertainties.period: period of observation; days: number of days analyzed;years: time span of observation.$\kappa_e \kappa_n \kappa_u$: estimated pectral index for GPS time series.

Baseline	v_e (mm/yr)	v_n (mm/yr)	σ_{v_e}(mm/yr)	σ_{v_n} (mm/yr)	v (mm/yr)	azimuth (degrees)
POBU-CAST	-0.173	0.044	0.122	0.062	0.179	-75.7
SJDV-TORI	-0.042	-0.090	0.109	0.072	0.099	-155.0
TENC-SAVI	-0.012	-0.184	0.096	0.050	0.184	-176.3
SAUV-MOND	0.189	-0.147	0.065	0.059	0.239	127.9
MTPL-GENO	-0.116	-0.007	0.077	0.026	0.116	-93.5
average	-0.050	-0.081	0.11	0.084	0.095	-148.3

Table 2: Relative velocities for selected baselines. Numbers indicates the velocity of the second site with respect to the first one. v is the norm of the velocity.

```
ROTATION RATE VECTOR
Wx (rad/yr): 4.5899E-10 +- 2.6941E-10
Wy (rad/yr): 7.9123E-11 +- 3.9879E-11
Wz (rad/yr): 4.6722E-10 +- 2.6709E-10
ASSOCIATED VARIANCE-COVARIANCE MATRIX (rad/yr)**2
        Wx        Wy          Wz
------------------------------------
Wx | 7.2582E-20 1.0711E-20 7.192E-20
Wy | 1.5904E-21 1.0615E-20
Wz | 7.1337E-20
------------------------------------
EULER POLE
longitude (dec. degree) : 9.78
latitude (dec. degree) : 45.09
angular velocity (deg/Myr ): 0.038
ASSOCIATED ERROR ELLIPSE
semi major axis : 0.79
semi minor axis : 0.44
azimuth of semi major axis : -175.2
std(angular velocity) : 0.022
STATISTICS
----------
Number of sites = 5
Chi**2 = 15.8
Deg. of. freedom = 7
A post. var. factor = 1.5
RESIDUALS
----------
site   R_ve   R_vn  S_ve  S_vn  RN_ve  RN_vn
-------------------------------------------------------------------------------------
CAST  -0.17   0.05  0.12  0.06  -1.37   0.83
GENO  -0.18  -0.01  0.08  0.03  -2.37  -0.29
MOND   0.11   0.02  0.07  0.06   1.65   0.41
SAVI   0.18  -0.05  0.10  0.05   1.82  -0.91
TORI  -0.03   0.05  0.11  0.07  -0.27   0.68
-------------------------------------------------------------------------------------
rms = 0.11 mm/yr wrms = 0.07 mm/yr
-------------------------------------------------------------------------------------
```

Table 3: Euler pole estimation for the best determined sites located in the western Po plain

```
ROTATION RATE VECTOR
Wx (rad/yr): -7.9581E-11 +- 1.6002E-10
Wy (rad/yr): 4.102E-12   +- 1.7584E-11
Wz (rad/yr): -1.4509E-10 +- 1.6111E-10
ASSOCIATED VARIANCE-COVARIANCE MATRIX (rad/yr)**2
        Wx          Wy          Wz
------------------------------------
Wx | 2.5606E-20 2.7997E-21 2.5777E-20
Wy | 3.0921E-22 2.8188E-21
Wz | 2.5956E-20
------------------------------------
EULER POLE
longitude (dec. degree) : 177.05
latitude (dec. degree) : -61.22
angular velocity (deg/Myr ): 0.009
ASSOCIATED ERROR ELLIPSE
semi major axis : 22.00
semi minor axis : 0.61
azimuth of semi major axis : -90.5
std(angular velocity) : 0.012
STATISTICS
----------
Number of sites = 16
Chi**2 = 1045.9
Deg. of. freedom = 29
A post. var. factor = 6.0
RESIDUALS
----------
site  R_ve  R_vn S_ve S_vn  RN_ve  RN_vn
------------------------------------------------------------------------------------------
ALPE -0.06  0.15 0.06 0.06  -0.99   2.58
BACT  0.37  0.03 0.11 0.12   3.28   0.28
CAST  0.12  0.04 0.12 0.06   1.01   0.61
CHAM  0.00 -0.19 0.04 0.05   0.08  -4.02
CHTL -0.28  0.16 0.02 0.02 -15.49   8.20
GRAV  0.82 -0.56 0.12 0.12   6.92  -4.84
GUIL  0.17 -0.01 0.10 0.14   1.81  -0.10
JANU  0.58 -0.70 0.06 0.06   9.82 -12.31
LFAZ  0.32  0.04 0.04 0.03   8.20   1.65
MODA  0.20 -0.20 0.03 0.03   7.97  -7.29
MRGE -0.24 -0.37 0.06 0.08  -4.28  -4.62
PUYA  0.63  0.02 0.07 0.06   8.81   0.36
RABU  0.60 -0.11 0.30 0.16   1.96  -0.66
ROSD -0.16 -0.36 0.13 0.12  -1.22  -2.91
STEY  0.42  0.09 0.10 0.07   4.05   1.34
VILR -0.13  0.28 0.07 0.04  -1.71   6.39
------------------------------------------------------------------------------------------
rms = 0.34 mm/yr wrms = 0.26 mm/yr
```

Table 4: Euler pole estimation for the western Alps

```
SITES USED IN CALCULATION
-------------------------
CHTL GRAV JANU MODA MRGE PUYA ROSD
VELOCITY GRADIENT
-----------------
dVx/dx dVx/dy dVy/dx dVy/dy
--------- -------- --------- --------
12.869 -12.618 -14.171 3.709
Unity : ppb / yr
STRAIN RATE TENSOR
------------------
Exx Exy Eyy
-------- -------- ---------
12.869 -13.394 3.709
Unity : ppb / yr
STRAIN RATE TENSOR COVARIANCE MATRIX(not normalized)
Exx Exy Eyy
Exx 0.64570E-09 -0.40376E-10 -0.37159E-25
Exy 0.33576E-09 0.72557E-11
Eyy 0.53983E-09
PRINCIPAL AXIS
Eps1 = 22.4447761015569 +- 0.831302996602003
Eps2 = -5.86671690707883 +- 0.770801861684347
Azimuth = 35.5619714641032 +- 1.08214127352853 Unity : ppb / yr and deg.dec
Eps1: most extensional eigenvalue of strain tensor
Eps2: most compressional eigenvalue of strain tensor Extension is taken positive azimuth is the one of e
strain tensor position (long. lat.) : 6.7091 45.2675
ROTATION
--------
rotation : 0.04449 +- 0.06640 deg / Myr
STATISTICS
----------
A POSTERIORI VARIANCE FACTOR IN VELOCITY GRADIENT LEAST-SQUARES ESTIMATION Vt.P.V : 72.50197
DEG. LIBERTE : 8 KI2 / DOF : 9.06275
RACINE(KI2/DOF) 3.01044
CHI2 TESTS OF SIGNIFICANCY
--------------------------
tested            chi2      threshold values
parameters        values    95%      99%
-----------------------------------------------
velocity gradient  772.43   9.49   13.28
strain rate tensor 761.53   7.82   11.35
rotation             0.45   3.84    6.64
```

Table 5: Strain rate tensor for the northwestern Alps

Supplementary Figures

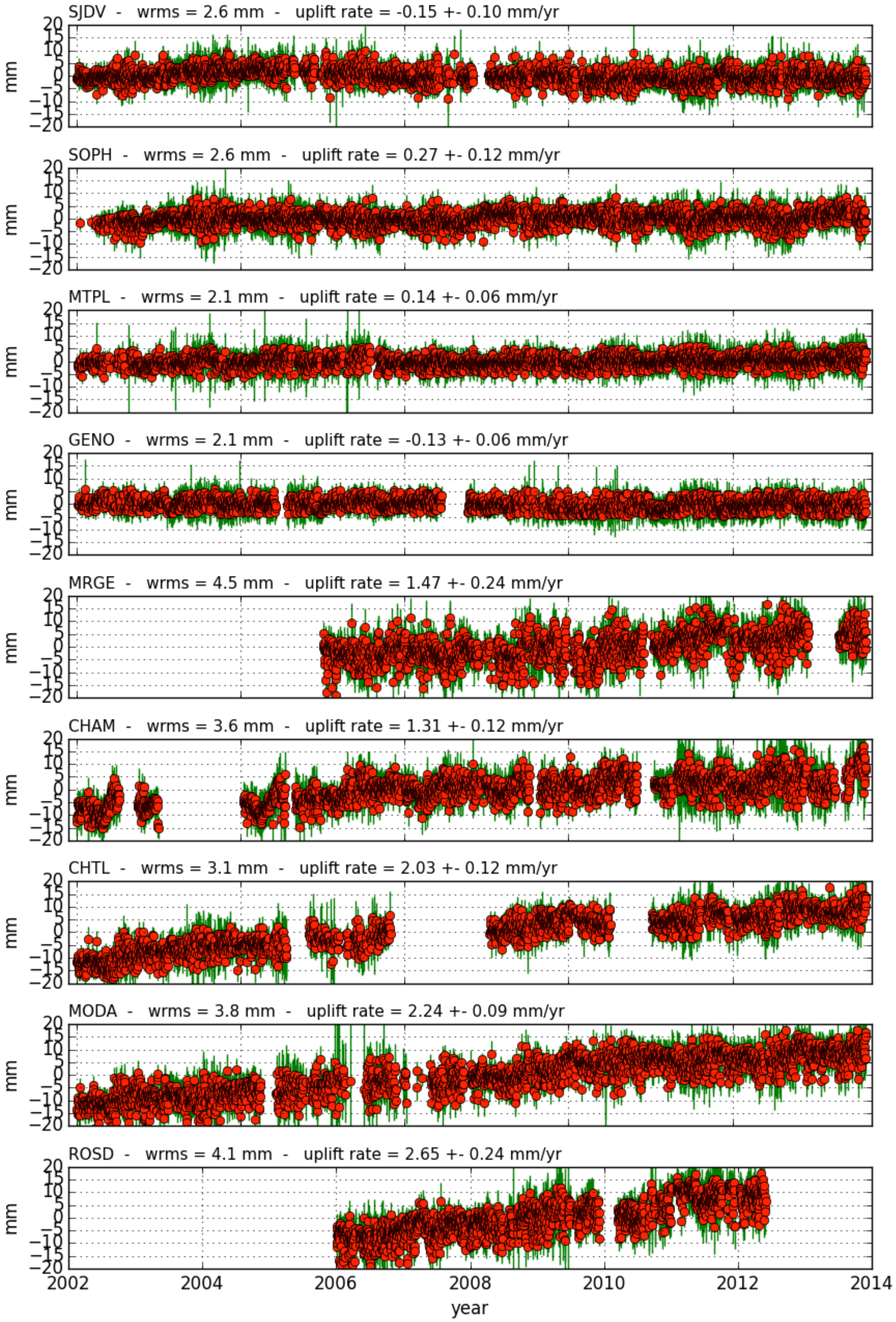

Supplementary Figure 1: Selection of some GPS times series (Up component). Figure created using Matplotlib v. 1.4 (http://matplotlib.org)

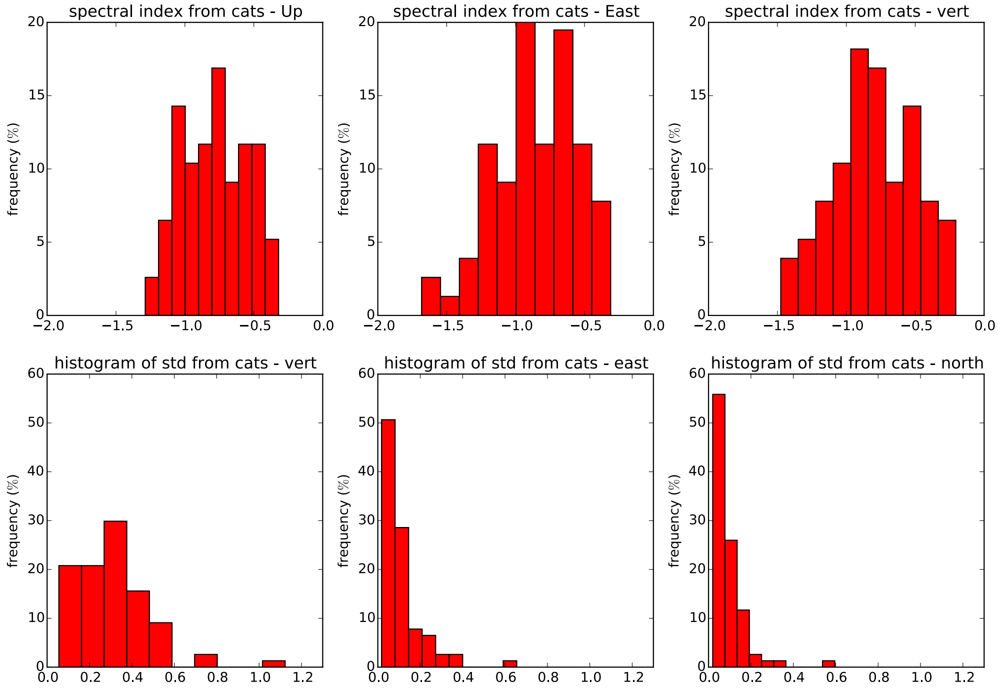

Supplementary Figure 2: Top: histograms of the spectral index characterizing the noise in the GPS time series, for the Up, East and North Components. Bottom: histograms of velocity uncertainties. Figure created using Matplotlib v. 1.4 (http://matplotlib.org)

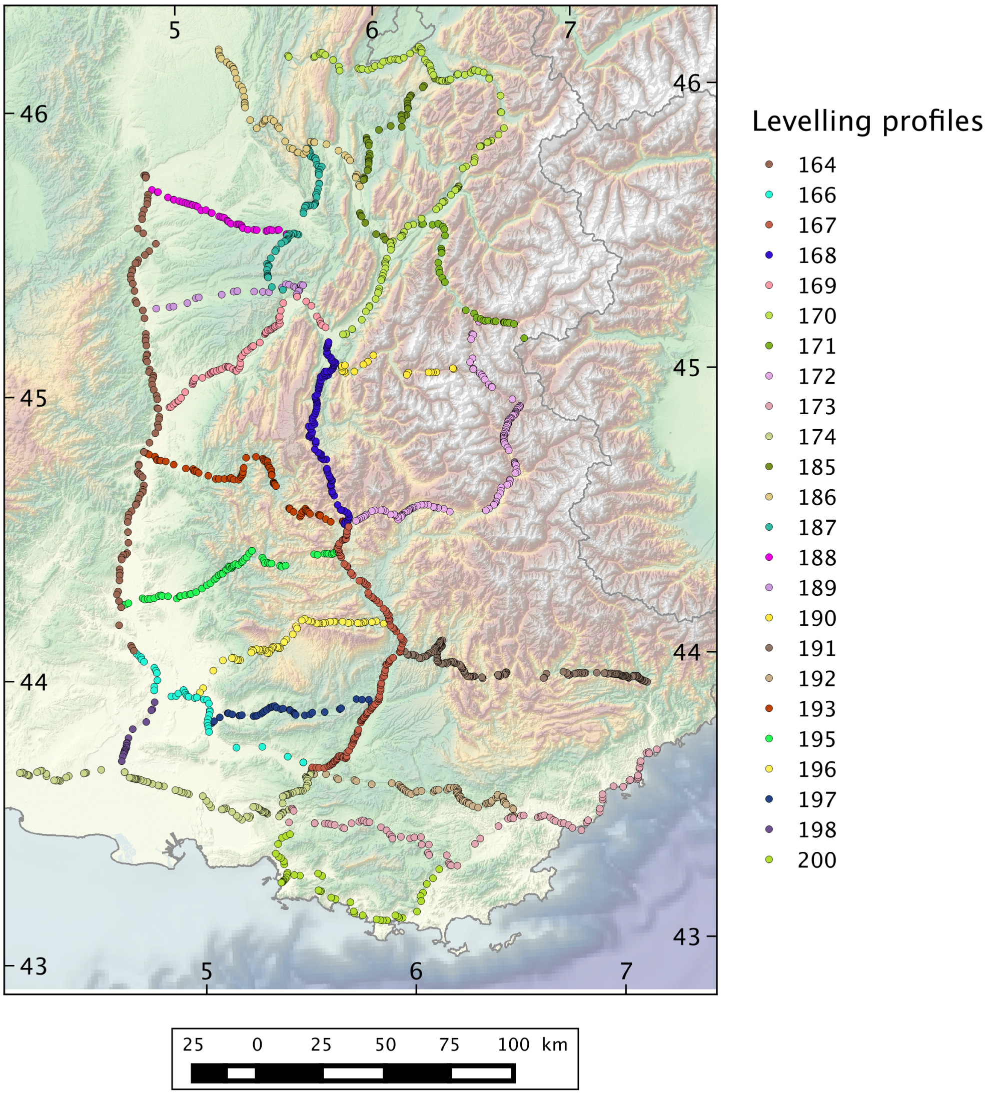

Supplementary Figure 3: Map of the levelling profiles used in the study. Figure created using QGIS v. 2.4 (http://www.qgis.org)

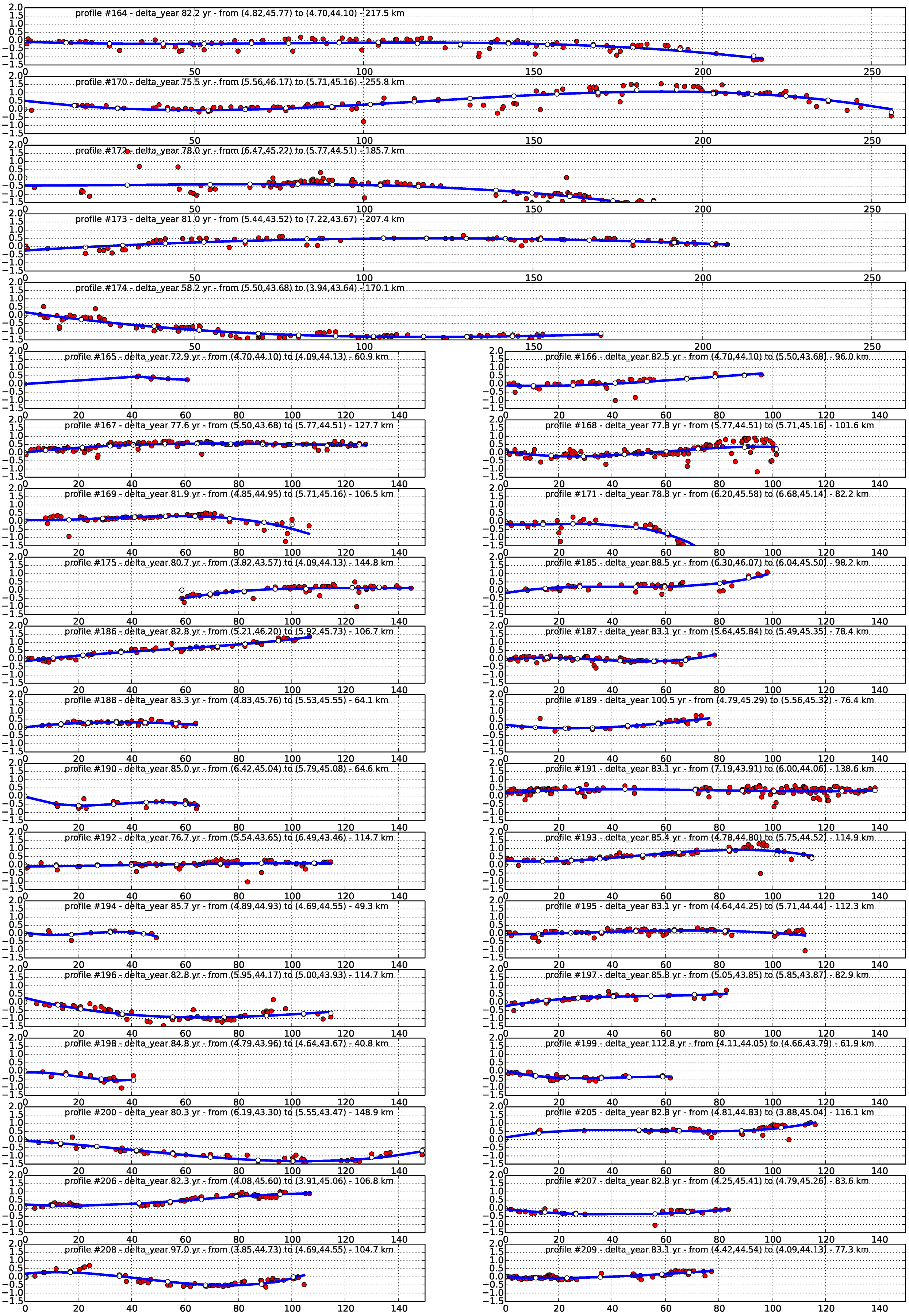
profile #164 - delta_year 82.2 yr - from (4.82,45.77) to (4.70,44.10) - 217.5 km
profile #170 - delta_year 75.5 yr - from (5.56,46.17) to (5.71,45.16) - 255.8 km
profile #172 - delta_year 78.0 yr - from (6.47,45.22) to (5.77,44.51) - 185.7 km
profile #173 - delta_year 81.0 yr - from (5.44,43.52) to (7.22,43.67) - 207.4 km
profile #174 - delta_year 58.2 yr - from (5.50,43.68) to (3.94,43.64) - 170.1 km
profile #165 - delta_year 72.9 yr - from (4.70,44.10) to (4.09,44.13) - 60.9 km
profile #166 - delta_year 82.5 yr - from (4.70,44.10) to (5.50,43.68) - 96.0 km
profile #167 - delta_year 77.6 yr - from (5.50,43.68) to (5.77,44.51) - 127.7 km
profile #168 - delta_year 77.8 yr - from (5.77,44.51) to (5.71,45.16) - 101.6 km
profile #169 - delta_year 81.9 yr - from (4.85,44.95) to (5.71,45.16) - 106.5 km
profile #171 - delta_year 78.8 yr - from (6.20,45.58) to (6.68,45.14) - 82.2 km
profile #175 - delta_year 80.7 yr - from (3.82,43.57) to (4.09,44.13) - 144.8 km
profile #185 - delta_year 88.5 yr - from (6.30,46.07) to (6.04,45.50) - 98.2 km
profile #186 - delta_year 82.8 yr - from (5.21,46.20) to (5.92,45.73) - 106.7 km
profile #187 - delta_year 83.1 yr - from (5.64,45.84) to (5.49,45.35) - 78.4 km
profile #188 - delta_year 83.3 yr - from (4.83,45.76) to (5.53,45.55) - 64.1 km
profile #189 - delta_year 100.5 yr - from (4.79,45.29) to (5.56,45.32) - 76.4 km
profile #190 - delta_year 85.0 yr - from (6.42,45.04) to (5.79,45.08) - 64.6 km
profile #191 - delta_year 83.1 yr - from (7.19,43.91) to (6.00,44.06) - 138.6 km
profile #192 - delta_year 76.7 yr - from (5.54,43.65) to (6.49,43.46) - 114.7 km
profile #193 - delta_year 85.4 yr - from (4.78,44.80) to (5.75,44.52) - 114.9 km
profile #194 - delta_year 85.7 yr - from (4.89,44.93) to (4.69,44.55) - 49.3 km
profile #195 - delta_year 83.1 yr - from (4.64,44.25) to (5.71,44.44) - 112.3 km
profile #196 - delta_year 82.8 yr - from (5.95,44.17) to (5.00,43.93) - 114.7 km
profile #197 - delta_year 85.8 yr - from (5.05,43.85) to (5.85,43.87) - 82.9 km
profile #198 - delta_year 84.8 yr - from (4.79,43.96) to (4.64,43.67) - 40.8 km
profile #199 - delta_year 112.8 yr - from (4.11,44.05) to (4.66,43.79) - 61.9 km
profile #200 - delta_year 80.3 yr - from (6.19,43.30) to (5.55,43.47) - 148.9 km
profile #205 - delta_year 82.8 yr - from (4.81,44.83) to (3.88,45.04) - 116.1 km
profile #206 - delta_year 82.3 yr - from (4.08,45.60) to (3.91,45.06) - 106.8 km
profile #207 - delta_year 82.8 yr - from (4.25,45.41) to (4.79,45.26) - 83.6 km
profile #208 - delta_year 97.0 yr - from (3.85,44.73) to (4.69,44.55) - 104.7 km
profile #209 - delta_year 83.1 yr - from (4.42,44.54) to (4.09,44.13) - 77.3 km

Supplementary Figure 4: Individual levelling profiles used as input data prior to adjustment. Red dots are the raw data. White dots indicate the sites used to sub-sample the levelling data for plots in the main text. The blue curve is a smooth spline fit of the raw data showing the long wavelength signal for each profile. Figure created using Matplotlib v. 1.4 (http://matplotlib.org)

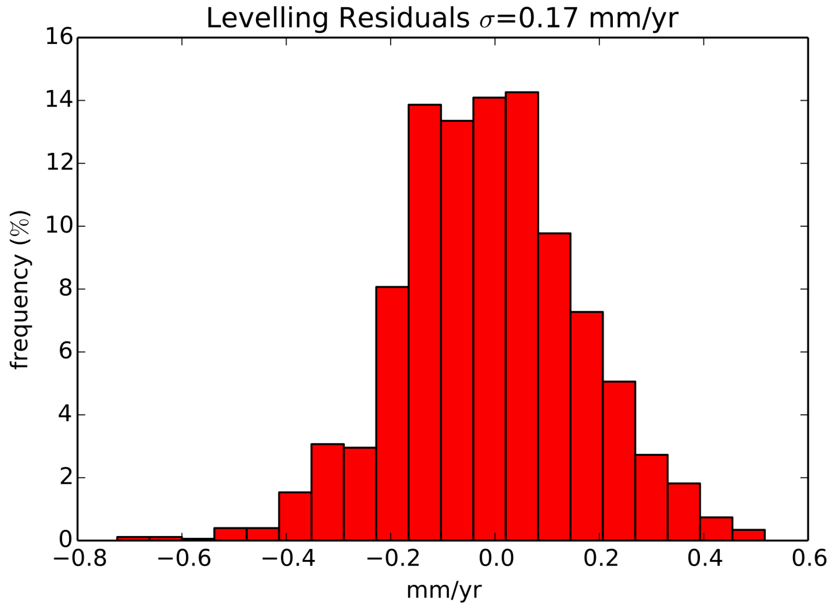

Supplementary Figure 5: Histogram of residuals in the levelling adjustment. Figure created using Matplotlib v. 1.4 (http://matplotlib.org)
